# The Effects of Aerobic Exercise on Oxidative Stress in Older Adults: A Systematic Review and Meta-Analysis

**DOI:** 10.3389/fphys.2021.701151

**Published:** 2021-10-05

**Authors:** Yu Ye, Huiying Lin, Mingyue Wan, Pingting Qiu, Rui Xia, Jianquan He, Jing Tao, Lidian Chen, Guohua Zheng

**Affiliations:** ^1^College of Rehabilitation Medicine, Fujian University of Traditional Chinese Medicine, Fuzhou, China; ^2^College of Nursing and Health Management, Shanghai University of Medicine & Health Sciences, Shanghai, China

**Keywords:** oxidative stress, aerobic exercise, older adults, review, meta-analysis

## Abstract

**Background:** Oxidative stress (OS) plays an important role in the progression of many aging-related diseases. Exercises can delay this kind of progress, but aerobic exercise is the most commonly used type of training among older adults; therefore, its influence needs to be further verified.

**Methods:** A literature search was conducted in eight electronic databases, including Cochrane, EMBASE, PubMed, Web of Science, China National Knowledge Infrastructure (CNKI), China Science and Technology Journal Database (VIP), Wanfang Date, and SinoMed from their inception to April 2020. Methodological quality was assessed using Cochrane RoB tool v2.0 for individual studies, and RevMan 5.3 software was used to perform the meta-analysis.

**Results:** The meta-analysis included 20 studies, involving 1,170 older adults. The results showed that regular aerobic exercise could reduce blood oxidant markers, including malondialdehyde (MDA; SMD=−1.80, 95% CI −2.46 to −1.14, *p*<0.001) and lipid peroxide (LPO; SMD=−1.12, 95% CI −2.03 to −0.22, *p*=0.02), and increase the levels of antioxidant factors, such as nitric oxide (NO; SMD=0.89, 95% CI 0.37–1.41, *p*<0.001), superoxide dismutase (SOD; SMD=0.63, 95% CI 0.25–1.01, *p*=0.001), and total antioxidant capacity (TAC; SMD=1.22, 95% CI 0.45–1.98, *p*=0.002), with clear statistical significance. It may also improve the levels of other OS markers, such as 8-OHdG, 8-isoPGF2, VE, and reduced glutathione/oxidized glutathione (GSH/GSSG).

**Conclusion:** Regular aerobic exercise may have a positive effect on the OS levels of older adults by reducing some oxidant markers and increasing antioxidant marker levels.

## Introduction

Aging is a dynamic, degenerative, biological, and time-dependent process. It is characterized by the gradual accumulation of damage to cells, progressive functional decline, and increased susceptibility to disease (Finkel and Holbrook, 2000). Therefore, aging is closely linked to the occurrence and development of a variety of age-related diseases, such as cancer, type 2 diabetes mellitus, and cardiovascular and neurodegenerative diseases (Wyss-Coray, 2016). Although the aging mechanisms are unclear, several theories have been proposed. Oxidative stress (OS) takes part in the development of aging, which has been proved to be associated with aging as well as agerelated diseases (Dröge, 2002; Campisi et al., 2019; Warraich et al., 2020). The levels of OS damage markers have been reported to increase in older adults, which is possibly associated with an uncontrolled production of free radicals [i.e., reactive oxygen species (ROS)] by aging mitochondria and decreased activity of antioxidant defenses (Andriollo-Sanchez et al., 2005). Evidence has shown a close relationship between aging and the deleterious and cumulative effects of ROS generated throughout the lifespan (Warraich et al., 2020).

Oxidative stress reflects an imbalance between oxidation and reduction. It is also understood as a disproportionate relationship between the oxidants and/or pro-oxidants and antioxidant molecules. This imbalance is also implicated in the pathophysiology of a variety of age-related diseases (Sies et al., 2017; Viña, 2019). ROS are generated as a byproduct of mitochondrial respiration or metabolism or by specific enzymes, and are highly reactive molecules that consist of diverse chemical species, including superoxide anion (O_2_^−^), hydroxyl radical (OH), hydrogen peroxide (H_2_O_2_), and peroxynitrite (OONO−; Szabó, 2003; Valko et al., 2006; Korovila et al., 2017). O_2_- can also react with nitric oxide (NO) to inactivate it; the OONO− formed by the reaction can oxidize NO synthase and make it unstable. And this unstable NO synthase will further increase the production of peroxide (Liguori et al., 2018). However, their formation can be neutralized by a suite of antioxidant compounds (e.g., reduced thiols, vitamins C and E, and catecholamines) and enzyme systems (e.g., superoxide dismutase, peroxidase, catalase, and glutathione reductase), which are synthesized in aerobic organisms in the body (Birben et al., 2012). OS occurs whenever ROS production is higher than antioxidant capacities, and is increased in older adults because of multiple factors, such as inadequate nutrient intake of proteins and vitamins, a sedentary lifestyle, declines in anabolic hormone levels, and increased levels of inflammatory cytokines, which lead to the production of ROS (Violi et al., 2017).

Exercise is a known strategy to modulate levels of OS in older adults, but the relationship between exercise and OS is extremely complex, depending on the mode, intensity, and duration of exercise. Studies have shown that regular exercise with light to moderate intensity could gradually strengthen endogenic antioxidant defense mechanism and diminish level of OS (De Sousa et al., 2017; Spanidis et al., 2018). Conversely, acute exercise and high-intensity exercise lead to increased level of OS (Pingitore et al., 2015). A significant number of studies have shown that, compared with single exercise (or acute exercise) and high-intensity exercise, regular aerobic exercise can activate the body’s antioxidant system, upregulate endogenous antioxidant factors, and strengthen the activity of antioxidant enzymes (Radak et al., 2008; Pillon Barcelos et al., 2017; Thirupathi and Pinho, 2018). Moreover, several studies have investigated the effects of aerobic exercise on OS, but the findings have been conflicting. For example, Thirupathi et al. (2020) found that running exercise did not affect the changes in OS markers. Another meta-analysis reported that any exercise intensity or pattern can increase or decrease oxidative damage and specific OS biomarkers (Thirupathi et al., 2021). The impact of exercise on human OS levels has been discussed in scientific research and clinical fields. Current research focuses on different types of exercise regimens, which target a wide range of individuals. Therefore, it is not certain which form or intensity of exercise can provide the most beneficial effects for older adults. The purpose of this review was to analyze the effect of regular aerobic exercise on OS markers in older adults.

## Materials and Methods

### Search Strategy

We searched eight databases, including Cochrane, EMBASE, PubMed, Web of Science, China National Knowledge Infrastructure (CNKI), China Science and Technology Journal Database (VIP), Wanfang Date, and SinoMed, from their inception to April 2020, without restrictions on language or publication type. The original search covered a combination of relevant keywords relating to aerobic exercise (e.g., “exercise,” “aerobic exercise,” and “endurance activity”), OS (e.g., “oxidative stress,” “oxidation reduction,” and “free radicals”), and older adults (e.g., “elderly,” “aging,” and “geriatric”). In addition, the references of all articles included in the study were checked to identify other relevant articles. The complete search strategy can be found in the Supplementary Material.

### Inclusion Criteria

Studies were included if: (1) the design of the study was a randomized controlled trial; (2) participants were older adults aged 60years old or older, no gender, or race restrictions; (3) the intervention in the experimental group could be any form of aerobic exercise (also known as endurance training and cardiopulmonary exercise) or aerobic exercise combined with nonexercise intervention measures (e.g., routine treatment, medication, nutritional supplements, and health education); however, aerobic exercise should occur at least three times a week, at least 20min each time and for no less than 4weeks; (4) the control group had either no specific exercise intervention (e.g., waiting for treatment and maintaining daily activity habits), or the same nonexercise intervention as the experimental group; and (5) outcome measures included one or more OS markers measured in serum or plasma.

### Study Selection and Data Extraction

All the searched records were imported into the reference management software (EndNote X9.3.2). After eliminating duplicated records using software, two researchers (YY and PTQ) independently checked and selected the eligible studies by reading the titles, abstracts, or full text based on the predefined eligible criteria. Any disagreements were resolved by discussion with the third reviewer (GHZ). Data in the eligible studies were extracted by one reviewer (YY) using the predesigned form and it was checked for accuracy by another reviewer (HYL). The extracted information included participants’ characteristics, study design, sample size, methodological information of study quality, aerobic exercise parameters (e.g., type, frequency, duration, and intensity of exercise), controls, outcomes and their measurement method, and adverse events.

### Assessment of Risk of Bias in Included Studies

Two reviewers (YY and MYW) independently assessed the risk of bias of the included studies using the revised Cochrane Collaboration’s risk of bias tool (RoB V.2.0) for RCTs (Sterne et al., 2019). The five recommended domains included bias arising from the randomization process, bias due to deviations from intended interventions, bias due to missing outcome data, bias in measurement of the outcome, and bias in selection of the reported result. The evaluation results of each domain for each study were categorized as “low risk of bias,” “some concerns,” or “high risk of bias” according to the answers to signaling questions. Discrepancies between two reviewers were resolved by discussion with the third reviewer (GHZ).

### Statistical Analysis

Before meta-analysis, inconsistent units of the same outcome were converted into uniform units, and outcomes that were not represented by the mean and SD were converted into the mean and SD. For studies with more than one eligible intervention group (i.e., different exercise training), we calculated the combined means and SD according to the Cochrane guidelines. The size of the effect from baseline to postintervention for each OS marker was expressed as the mean difference (MD) and its 95% CI if the measurement method was the same; if not, the standardized MD and its 95% CI were used (Morris, 2007). The heterogeneity among the included studies was evaluated using the Chi-square test, tau-squared, and Higgins I^2^ value. With the Chi-square test, *p*<0.05 was considered to be significant. If there was no significant heterogeneity among studies, the pooled effect was calculated using the fixed-effect model. Otherwise, the random-effects model was applied (Higgins et al., 2003). The reasons for heterogeneity were examined by subgroup analysis if possible. A funnel plot was used to assess the publication bias if there were more than 10 studies with the same outcome. A leave-out-one sensitivity analysis was conducted by excluding each study at a time to identify the impact of each study on the overall estimate. A two-sided value of *p*<0.05 was regarded as statistically significant. All the analyses were conducted using Review Manager 5.4 software. The outcomes with missing data were analyzed using the narrative synthesis approach.

## Results

### Study Selection

**Figure 1:**
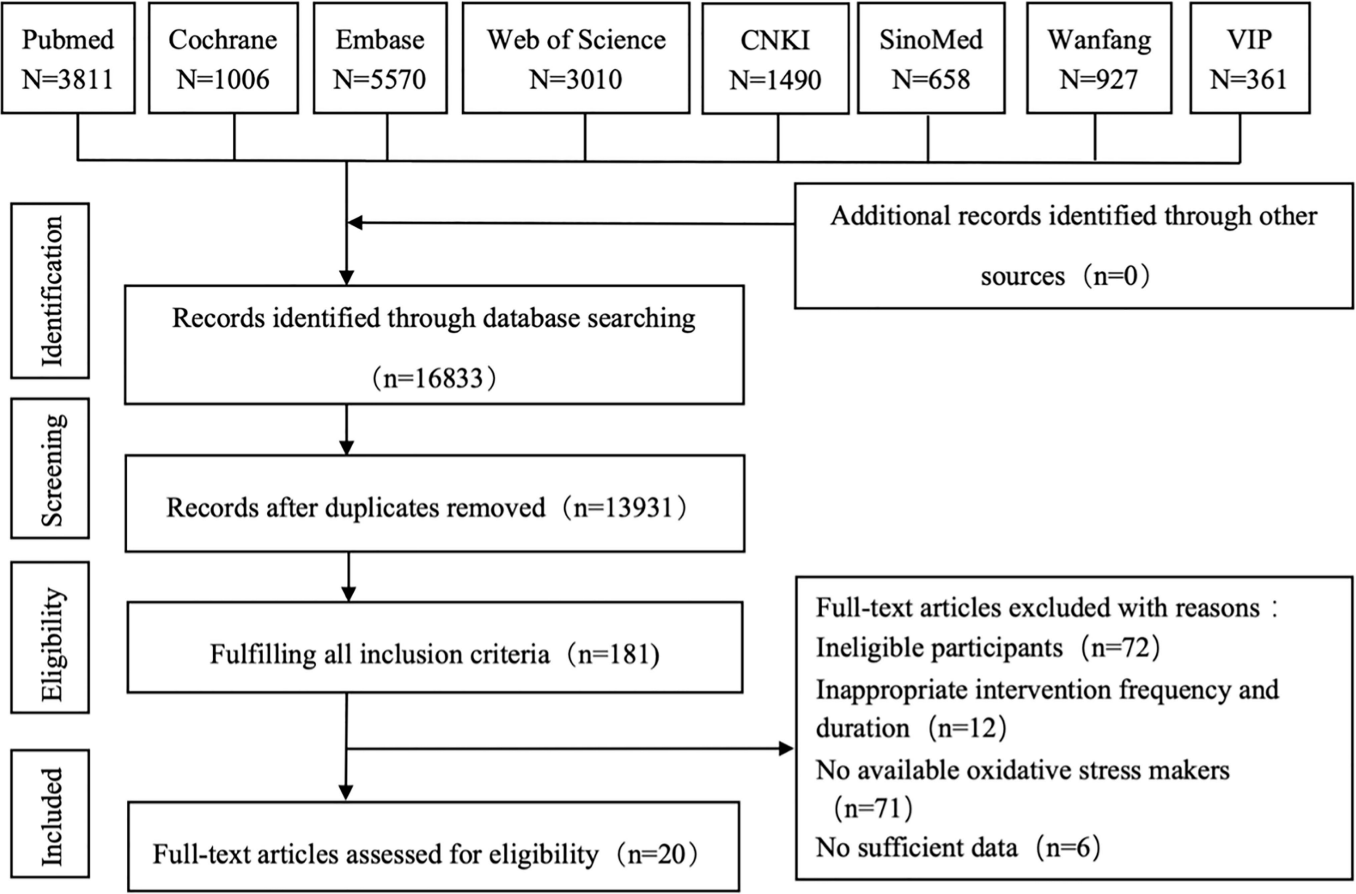
Flow diagram for the strategy of searching for the studies. CNKI, China National Knowledge Infrastructure; VIP, China Science and Technology Journal Database.

According to the search strategy, 16,833 citations were retrieved from the eight databases. After removing duplicates, two reviewers (YY and LHY) screened titles and abstracts to exclude unrelated records. Then, 181 full-text articles were assessed for eligibility according to the inclusion criteria. Finally, 20 studies involving 1,170 older adults were included in the meta-analysis (Shi et al., 1995; Liu et al., 1996; Yao et al., 2001; Fatouros et al., 2004; Sha, 2007; Zhu et al., 2008; Cetin et al., 2010; Liu and Jin, 2010; He et al., 2011; Xie and Bai, 2014; Zhen et al., 2014; Groussard et al., 2015; Wang et al., 2015; Xiong and Feng, 2015; Yang et al., 2015; Alghadir et al., 2016; Ma et al., 2017; Mohammadi et al., 2018; Hejazi et al., 2019; Osali, 2020). The detailed flow diagram is shown in Figure 1.

### Study Characteristics

There were 20 randomized controlled trials involving 1,170 older adults, including 546 male subjects and 624 female subjects, with an average age of 62.3–72.4years old. The types of aerobic exercise used in the experimental group were diverse, most of which were walking (Shi et al., 1995; Cetin et al., 2010; Xiong and Feng, 2015) or combined walking with jogging (Fatouros et al., 2004; Ma et al., 2017; Osali, 2020), jogging (Shi et al., 1995), cycling (Groussard et al., 2015; Yang et al., 2015; Alghadir et al., 2016), Tai Chi (Shi et al., 1995; Liu et al., 1996; Sha, 2007; Xie and Bai, 2014), Baduanjin (He et al., 2011; Zhen et al., 2014; Wang et al., 2015), Wu Qin Xi (Zhu et al., 2008), and Yi Jinjing (Liu and Jin, 2010). Three studies did not describe specific exercise programs (Yao et al., 2001; Mohammadi et al., 2018; Hejazi et al., 2019). The frequency of exercise intervention was between 35 and 60min per session with 3–7 sessions per week for 4weeks to 3years. Exercise intensity was controlled at 40–80% of the stored maximum heart rate (Shi et al., 1995; Yao et al., 2001; Fatouros et al., 2004; Cetin et al., 2010; Liu and Jin, 2010; Xie and Bai, 2014; Groussard et al., 2015; Yang et al., 2015; Alghadir et al., 2016; Ma et al., 2017; Mohammadi et al., 2018; Hejazi et al., 2019; Osali, 2020), two articles described it as “medium intensity” (Zhu et al., 2008; He et al., 2011), and five articles did not mention exercise intensity (Liu et al., 1996; Sha, 2007; Zhen et al., 2014; Wang et al., 2015; Xiong and Feng, 2015). All controls were set to no specific exercise intervention or the same conventional treatment as the experimental group. The measured OS markers included six oxidant markers [i.e., malondialdehyde (MDA), 8-hydroxy-2'-deoxyguanosine (8-ODdG), oxidized low-density lipoprotein (Ox-LDL), etc.] and seven antioxidant markers [i.e., total antioxidant capacity (TAC), reduced glutathione/oxidized glutathione (GSH/GSSG), superoxide dismutase (SOD), etc.], but their measurement methods were widely diverse. The characteristics of included clinical trials are described in Table 1.

**Table 1:** Characteristics of studies included in the present study.

Author, year	Participants	Mean age	Participants (M/F)	Intervention	Frequency, duration, and intensity of aerobic exercise	Outcomes/measure
Cetin et al., 2010	Healthy elderly	70.3	29 (24/5)	T: walking C: no training	50min/day, 3days/week, 6months, and 60–75% HRmax	VE/Spectrofluorometrically TAC/Colorimetric assay kit
Mohammadi et al., 2018	Overweight elderly men	72.4	24 (24/0)	T: aerobic exercises C: maintain their usual lifestyle	45–60min/day, 3days/week, 8weeks, and 90–95% HRmax	NO/Zebra Kit
Hejazi et al., 2019	Elderly healthy women	65.5	21 (0/21)	T: aerobic exercises C: maintain their usual lifestyle	45–60min/day, 4days/week, 8weeks, and 50–70% HRmax	8-OHdG/ELISA
Groussard et al., 2015	Adults with ESKD	67.6	18 (13/5)	T: specialized cycle ergometers C: no training	30min/day, 3days/week, 3months, and 50–60% HRmax	Ox-LDL/ELISA GSH/GSSG/GSH/GSSG-412 kit SOD/Ransod Kit GPX/Ransel Kit
Alghadir et al., 2016	Healthy elderly	69.7	100 (70/30)	T: treadmill, bicycle, and StairMaster C: no training	45–60min/day, 3days/week, 24weeks, and 60–70% HRmax	TAC/Colorimetric Assay Kit MDA/HPLC 8-OHdG/ELISA
Fatouros et al., 2004	Elderly healthy man	72.3	19 (19/0)	T: walking/jogging C: no training	60min/day, 3days/week, 16weeks, and 50–80% HRmax	MDA/TBA 3-NT/HPLC TAC/Chemiluminescence GPX/Hitachi 2001 UV/VIS spectrophotometer
Osali, 2020	Women with metabolic syndrome	62.3	22 (0/22)	T: walking/running C: no training	36–51min/day, 7days/week, 6weeks, and 65–75% HRmax	TAC/The ferric reducing ability of plasma MDA/Esterbauer and Cheeseman method
Xiong and Feng, 2015	Healthy elderly	63.4	40 (20/20)	T: walking C: no training	35–45min/day, 60days	SOD, GPX, GSH, MDA
Liu et al., 1996	Elderly healthy man	64.8	86 (86/0)	T: Tai Chi C: no training	60min/day, 5days/week, and 1year	SOD/Catechol Autoxidation LPO/TBA colorimetry
Sha, 2007	Adults with AS	64.1	40 (24/16)	T: Tai Chi C: no training	60–120min/day, 5–7days/week, and 18months	NO/NR
Xie and Bai, 2014	Adults with EH	60–70	50 (25/25)	T: Tai Chi C: no training	60min/day, 5days/week, 12weeks, and 40–60% HRmax	NO/NR
Shi et al., 1995	Adults with EH	68.2	32 (25/7)	T: walking/jogging/Tai Chi C: no training	60min/day, 3years, and 70% HRmax	LPO/TBA
Yao et al., 2001	Adults with hypertension	65.8	41 (10/31)	T: aerobic exercises C: no training	60min/day, 6days/week, 7weeks, and 50–60% HRmax	NO/NR
Zhu et al., 2008	Elderly healthy women	63	75 (0/75)	T: Wu Qin Xi C: no training	45min/day, 16weeks, and moderate intensity	SOD/XO MDA/TBA
He et al., 2011	Elderly healthy women	62.8	80 (0/80)	T: Baduanjin C: no training	45min/day, 20weeks, and moderate intensity	SOD/XO MDA/TBA
Zhen et al., 2014	Adults with EH	69.6	55 (29/26)	T: conventional treatment and Baduanjin C: conventional treatment	30min/day, 5days/week, and 12weeks	NO/NR
Liu and Jin, 2010	Elderly healthy women	65.7	62 (0/62)	T: Yi Jinjing C: no training	40–50min/day, 6days/week, 6months, and 60–75% HRmax	SOD/Hydroxylamine oxidation GPX/DTNB direct colorimetry MDA/TBA colorimetry
Wang et al., 2015	Adults with T2DM	64.2	100 (45/55)	T: Baduanjin C: no training	60min/day, 3days/week, and 1year	8-isoPGF2/ELISA SOD/Fluorescence
Yang et al., 2015	Adults with ISH	68.1	166 (59/107)	T: conventional treatment and bicycling C: conventional treatment	30min/day, 3days/week, 20weeks, and 60–80% HRmax	NO/Greiss
Ma et al., 2017	Adults with CHF	61–78	110 (73/37)	T: conventional treatment and combination of aerobic activity C: conventional treatment	30–40min/day, 4days/week, 12weeks, and 60–80% HRmax	NO/NR

VE, vitamin E; TAC, total antioxidant capacity; NO, nitric oxide; 8-OHdG, 8-hydroxy-2'-deoxyguanosine; ELISA, enzyme linked immunosorbent assay; ESKD, end-stage kidney disease; Ox-LDL, oxidized low-density lipoprotein; GSH/GSSG, reduced glutathione/oxidized glutathione; SOD, superoxide dismutase; GPX, glutathione peroxidase; MDA, malondialdehyde; HPLC, high performance liquid chromatography; TBA, thiobarbituric acid; 3-NT, 3-Nitrotyrosine; GPX, glutathione peroxidase; GSH, glutathione; LPO, lipid peroxide; AS, atherosclerosis; NR, nitrate reductase; EH, essential hypertension; XO, xanthine oxidase; DTNB, 5,5'-Dithiobis (2-nitrobenzoic acid); T2DM, type 2 diabetes mellitus; 8-isoPGF2, 8-iso-prostaglandin F2α; ISH, isolated systolic hypertension; and CHF, coronary heart disease with chronic heart failure.

### Risk of Bias Within Studies

**Figure 2:**
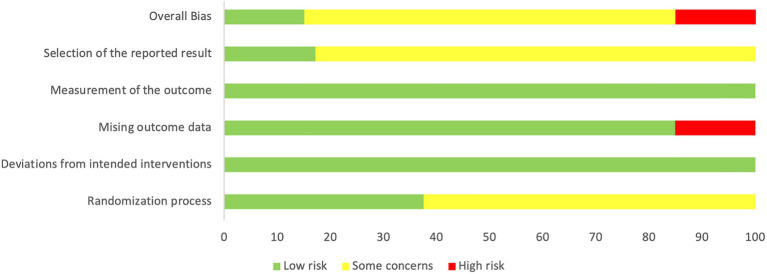
Percentage distribution of the risk of bias in individual studies (RoB 2).

**Figure 3:**
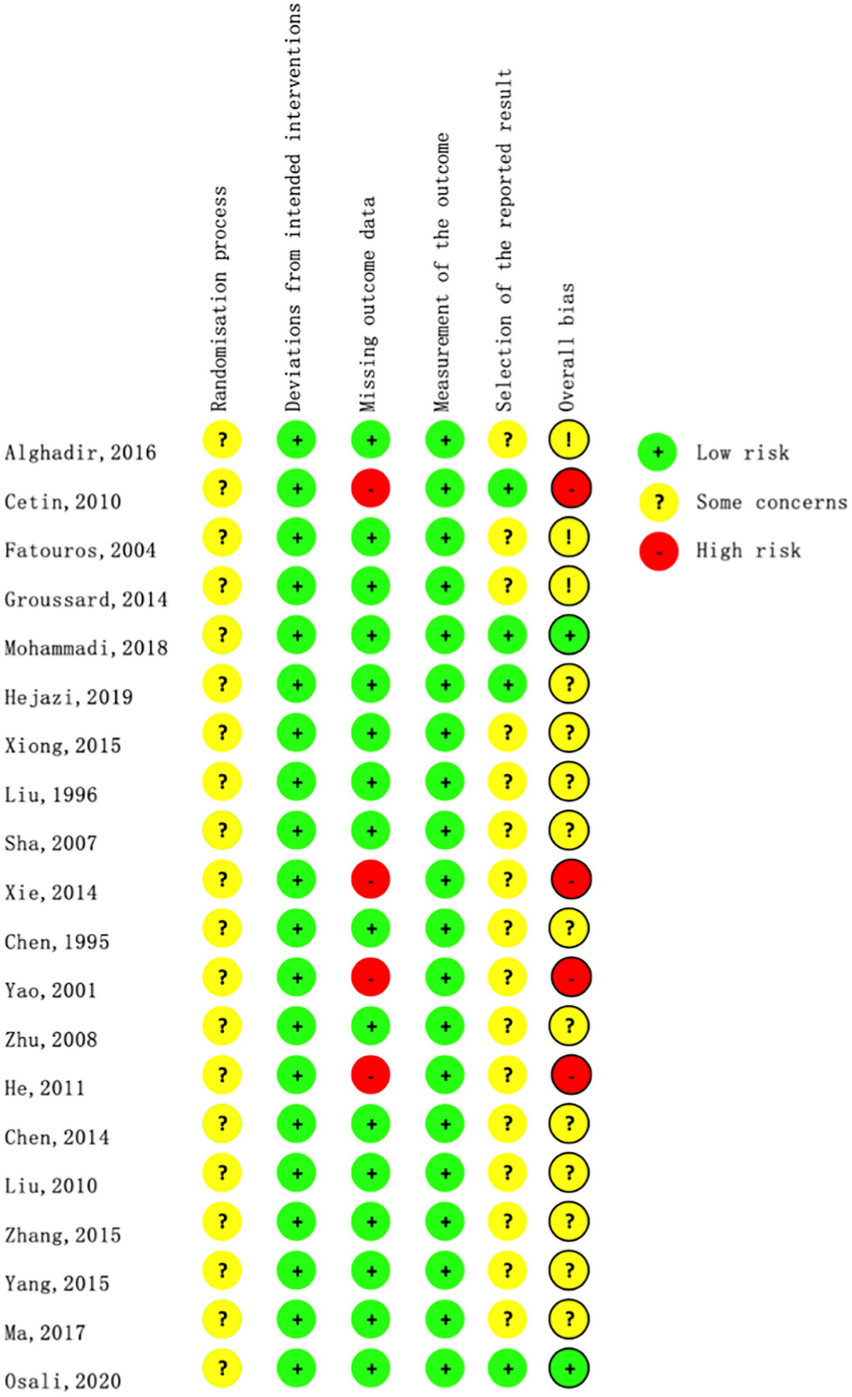
Risk of bias summary.

Figure 2 shows the percentage distribution of the risk of bias in individual studies (RoB 2). A total of 5.1% of the trials were rated as having a low risk of bias, 69.9% were rated as having some concerns, and 15.1% were rated as having a high risk of bias. A total of 62.4% of the studies did not adequately describe the randomized method, and only three of them described the method of randomization sequence generation by using random number tables (Sha, 2007; Zhen et al., 2014; Ma et al., 2017). Three studies explicitly reported allocation concealment (Zhen et al., 2014; Mohammadi et al., 2018; Osali, 2020). One study had a dropout rate of more than 20% (Cetin et al., 2010), and the other three did not mention missing data (Yao et al., 2001; He et al., 2011; Xie and Bai, 2014); thus, they were rated as high risk in the bias due to missing outcome data. Only four studies had preregistered protocols (Cetin et al., 2010; Mohammadi et al., 2018; Hejazi et al., 2019; Osali, 2020), and 82.8% of the studies therefore had some concerns regarding bias in the selection of the reported results. Figure 3 presents a summary of the risk of bias of the included studies.

### Effect of Interventions

#### Results of Individual Study of Oxidant Markers

Seven studies investigated the effect of aerobic exercise on MDA. Meta-analysis showed that aerobic exercise led to a significant decrease compared with the controls, with high heterogeneity across studies (SMD=−1.80, 95% CI −2.46 to −1.14, *p*<0.001, *I*^2^ =86%, seven studies, 398 samples). Subgroup analysis based on exercise type showed that both body–mind exercise and other aerobic exercise types could significantly decrease the MDA levels. Furthermore, no particular study prominently affected the overall effects by the sensitivity analysis (Table 2). Other oxidant markers, including 8-OHdG, lipid peroxide (LPO), 3-nitrotyrosine (3-NT), Ox-LDL, and 8-iso-prostaglandin F2α (8-isoPGF2), were measured in two or one included study. We observed a significant decrease in the levels of LPO, 8-OHdG, and 8-isoPGF2 in the aerobic exercise intervention group compared with the control group (SMD-LPO=−1.12, 95% CI −2.03 to −0.22, *p*=0.02, *I*^2^ =74%, two studies, 117 samples; MD-8-OHdG=−20.78, 95% CI −21.87 to −19.68, *p*<0.001, *I*^2^ =50%, two studies, 121 samples; and MD-8-isoPGF2=−98.92, 95% CI −147.81 to −49.83, one study, 87 samples); no significant differences were found in the levels of 3-NT or Ox-LDL markers between the two groups (MD-3-NT=−0.20, 95% CI −0.59–0.19, *p*=0.31, one study, 19 samples; MD-Ox-LDL=−2.0, 95% CI −5.65–1.65, one study, 18 samples).

**Table 2:** Meta-analysis of the comparison of oxidant markers between aerobic exercise and control group.

Marker	Analysis	Subgroup type	Studies	Samples I/C	Test for total effect			Heterogeneity
					MD/SMD	95% CI	*p*	*I*^2^ (%)
MDA	All		7	209/189	−1.80	−2.46, −1.14	<0.001	86
	Subgroup	Mind–body exercise	3	117/100	−2.07	−3.61, −0.53	0.008	95
		Other aerobic exercise type	4	92/89	−1.63	−1.97, −1.29	<0.001	0
8-OHdG	All		2	61/60	−20.78^*^	−21.87, −19.68	<0.001	50
LPO	All		2	60/57	−1.12	−2.03, −0.22	0.02	74

I, intervention group; C, control group; MD, mean difference; SMD, standardized mean difference; CI, confidence interval; MDA, malondialdehyde; 8-OHdG, 8-hydroxy-2'-deoxyguanosine; and LPO, lipid peroxide.

* Intervention effect reported as MD.

#### Results of Individual Study of Antioxidant Markers

Seven studies investigated NO levels in plasma or serum. The pooled results showed a significant increase in the aerobic exercise group compared with the control group (SMD=0.89, 95% CI 0.37–1.41, *p*<0.001, seven studies, 486 samples), with obvious heterogeneity (*I*^2^ =83%). Subgroup analysis based on different exercise types (mind–body exercise or other aerobic exercise types) or intervention methods (aerobic exercise alone or aerobic exercise combined with conventional treatment) also showed a significant improvement in the NO level, except for the subgroup of aerobic exercise interventions alone (SMD=0.54, 95% CI −0.17–1.25, four studies, 155 samples). However, the sensitivity analysis for the subgroup of aerobic exercise interventions alone showed a significant improvement when the study by Yao et al. (2001) was removed (SMD=0.82, 95% CI 0.36–1.28, three studies, 114 samples). The pooled SOD and TAC levels from seven studies and four studies, respectively, also showed a significant increase in the aerobic exercise intervention group compared with the controls (SMD-SOD=0.63, 95% CI 0.25–1.01, *p*=0.001, *I*^2^ =72%, seven studies, 448 samples; SMD-TAC=1.22, 95% CI 0.45–1.98, *p*=0.002, *I*^2^ =74%, four studies, 164 samples). Subgroup analysis of SOD based on different exercise types (the mind–body exercise or other aerobic exercise types) showed that mind–body exercise, but other aerobic exercise types had a significant effect on improving the SOD levels (Table 3). No significant difference was found in the glutathione peroxidase (GPX) levels (SMD=0.68, 95% CI −0.31–1.67, *p*=0.18, *I*^2^ =85%, four studies, 139 samples) or glutathione (GSH) levels (MD=0.16, 95% CI −0.15–0.47, *p*=0.30, one study, 40 samples) between groups. In addition, one study reported a significant increase in vitamin E levels (MD=384.00, 95% CI 330.91–437.09, *p*<0.001, one study, 23 samples), but another study found a significant decrease in GSH/GSSG levels (MD=−155.00, 95% CI −275.57 to −34.43, *p*=0.01, one study, 18 samples) in the aerobic exercise intervention group compared with the control group.

**Table 3:** Meta-analysis of the comparison of antioxidant markers between aerobic exercise and control group.

Marker	Analysis	Subgroup type	Studies	Samples I/C	Test for total effect			Heterogeneity
					MD/SMD	95% CI	*p*	*I*^2^ (%)
NO	All		7	253/233	0.89	0.37, 1.41	<0.001	83
	Subgroup	Mind–body exercise	3	72/73	0.68	0.34, 1.01	<0.001	0
		Other aerobic exercise type	4	181/160	1.04	0.18, 1.90	0.02	90
	Subgroup	Aerobic exercise combined with conventional treatment	3	163/168	1.29	0.68, 1.89	<0.001	82
		Aerobic exercise alone	4	90/65	0.54	−0.17, 1.25	0.14	75
SOD	All		7	203/218	0.63	0.25, 1.00	0.001	72
	Subgroup	Mind–body exercise	5	202/188	0.73	0.31, 1.14	<0.001	74
		Other aerobic exercise type	2	28/30	0.22	−0.94, 1.38	0.71	76
GPX	All		4	71/68	0.68	−0.31, 1.67	0.18	85
TAC	All		4	83/81	1.22	0.45, 1.98	0.002	74

I, intervention group; C, control group; MD, mean difference; SMD, standardized mean difference; CI, confidence interval; NO, nitric oxide; SOD, superoxide dismutase; GPX, glutathione peroxidase; and TAC, total antioxidant capacity.

### Adverse Events

No adverse events related to aerobic exercise intervention were reported in the included studies.

## Discussion

### Summary of Evidence

This systematic review and meta-analysis were based on 20 RCTs to explore the effects of regular aerobic exercise interventions on OS markers in older adults. The aggregated data show that aerobic exercise intervention significantly decreased the levels of blood oxidant markers, including MDA, LPO, 8-OHdG, and 8-isoPGF2, and increased the levels of antioxidant markers, such as TAC, NO, SOD, vitamin E, and GSH/GSSG, compared to the controls. These findings indicated that regular aerobic exercise intervention had a positive effect on OS in older adults. The included studies did not report any adverse events related to aerobic exercise intervention in this review, indicating that it is safe for older adults.

Aging leads to the steady accumulation of detrimental cellular and molecular changes within tissues. Oxidative damage may result in telomere attrition that accelerates aging. In addition, it is well known that mitochondria are the main sources of free radical production and are negatively affected by aging, leading to their inability to adapt to higher levels of OS. Exercise can increase resistance against OS in the brain or other issues and downregulates the levels of the oxidative damage, but this effect depends on the type of exercise used (Tuon et al., 2012). Regular exercise can induce mitochondrial adaptation and improve mitochondrial plasticity by attenuating their deficits, restoring their turnover, and promoting a healthier mitochondrial pool (Joseph et al., 2016). Aerobic exercise is accompanied by increased VO_2_, which may increase free radical activity. As exercise intensity increases, free radical production and OS also increase. However, antioxidant capacity is not overreached, and free radical-induced damage does not appear when the intensity of aerobic exercise is less than 75% VO_2max_ (Cooper et al., 2002). A systematic review and meta-analysis of 19 studies showed that regular moderate aerobic exercise might promote antioxidant capacity in the brain, but aerobic exhausted exercise, anaerobic exercise, or combination exercise may deteriorate the antioxidant response (Camiletti-Moirón et al., 2013). Due to the limitation of physical conditions, most older adults are engaged in low-moderate intensity aerobic exercise (Lee et al., 2017). The exercise intensity of the included studies in the present review was assessed by maximum heart rate (HR_max_), and most of them ranged from 40 to 80% HR_max_; thus, they belong to low-moderate intensity with VO_2max_ less than 75% (Londeree and Ames, 1976). Therefore, it is reasonable that our review suggests a significant improvement in regular aerobic exercise for the OS of elderly participants.

Oxidative stress occurs when there is an imbalance between ROS formation and the antioxidant defense systems in favor of pro-oxidant processes (Nocella et al., 2018). Moreover, OS also plays an important role in various aging-related diseases (Valko et al., 2007). When the pro-oxidant markers present in the blood exceed normal physiological levels, they can cause oxidative damage (Andican et al., 2012; Halliwell, 2012). The lipid peroxidation reaction of biological membranes caused by free radicals can cause unsaturated fatty acid peroxide and MDA, which leads to errors in DNA replication and body aging (Frijhoff et al., 2015; Lichtenberg and Pinchuk, 2015). Plasma LPO can be initiated by a variety of oxidants, including H_2_O_2_, superoxide, and other oxidants, and increases as a result of the reduced antioxidant defense system. As an end-product of lipid peroxidation, MDA can be produced by oxidized lipid induction. Therefore, MDA is one of the most frequently used indicators of pro-oxidant markers (Ramana et al., 2013). In our review, most of the included studies measured MDA levels as the main indicators of pro-oxidants. Other indicators of pro-oxidants, such as LPO, 8-OHdG, Ox-LDL, and 3-NT, were measured in one or two included studies. In fact, exercise induced an increase in the production of ROS, which could activate the body’s antioxidant defense system. After a certain period of regular exercise, repeated training stimulated mitochondrial biogenesis and also seemed to involve reconstruction (Perry et al., 2010; Little and Cochran, 2011). The findings in the present review show that regular aerobic exercise greatly improves the efficiency of enzymatic and nonenzymatic antioxidant defense systems and strengthens the body’s ability to remove pro-oxidants. In agreement with our reviews, previous research has also reported that regular aerobic exercise can reduce pro-oxidants, including MDA, LPO, and Ox-LDL levels (Brinkley et al., 2011; Rytz et al., 2020).

Nuclear factor kappa Β (NF-kΒ), a transcription factor that is modified by the redox state of the cell, participates in cellular responses to OS status and ROS-producing agents (Xiao et al., 2018), and inhibition of NF-kB can prevent oxidative modification of ROS-producing agents, which postpone the progression of atherogenesis (Çevikelli-Yakut et al., 2020). Aerobic exercise can inhibit the activation of human T cell NF-kB by reducing ROS-producing agents, such as tumor necrosis factor –α (TNF-α; Larsen et al., 2001). NO can directly modify the sulfhydryl residues of proteins through S-nitrosylation, preventing cells from being further oxidized by ROS (Sun et al., 2006). High levels of ROS can quickly inactivate bioactive NO to form cytotoxic OONO−, inhibit or decompose NO synthase, and promote vascular OS (Förstermann, 2010). Aerobic exercise reduces oxidative stress by increasing the bioavailability of NO, thereby effectively preventing the occurrence of hypertension and cardiovascular disease (Korsager Larsen and Matchkov, 2016). SOD represents a group of enzymes that catalyze the dismutation of O2^−^ and the formation of H_2_O_2_, which is the major defense against superoxide radicals (Bresciani et al., 2015). Numerous studies in animals and humans have shown that aerobic exercise increases antioxidant enzyme activity, such as SOD and GPX, in the body or in tissues. In addition, aerobic exercise can modify the levels of nonenzymatic antioxidants (Finaud et al., 2006). A clinical study found that aerobic exercise treatment could reverse age-induced endothelial dysfunction by activating the nitric oxide–cyclic guanosine monophosphate pathway and by upregulating SOD in the human body (Jarrete et al., 2014). TAC may provide more biologically relevant information than individual antioxidants. Plasma TAC is modulated mainly by radical overload, and it can be considered as more representative of the *in vivo* balance between oxidizing species and antioxidants (Ghiselli et al., 2000). Several studies have shown that regular aerobic exercise with 75–80% heart rate reserve intensity for 4–6weeks could improve total antioxidant capacity in young college students and male soccer athletes (Diaz et al., 2011; Yol et al., 2020). In our review, the pooled results showed that regular aerobic exercise significantly increased plasma antioxidant markers, including NO, TAC, and SOD levels. These findings provide support for regular aerobic exercise to improve the efficiency of enzymatic and nonenzymatic antioxidant defense systems.

### Strengths and Limitations

To the best of our knowledge, this is the first systematic review and meta-analysis to investigate the impact of regular aerobic exercise on OS levels in older adults. Most previous studies were narrative reviews and focused on exercise involving a very broad exercise type (aerobic, anaerobic, and resistance; Bloomer and Goldfarb, 2004; Pingitore et al., 2015; Chico et al., 2017). According to the American College of Sports Medicine, regular aerobic exercise with moderate intensity is the most suitable for older adults to promote their health (Chodzko-Zajko et al., 2009). This systematic review focused on the effect of regular aerobic exercise with low-moderate intensity on OS among older adults, and the findings should be valuable for older adults. Compared with young people, the change in OS level is more apparent in older adults (Konopka and Sreekumaran Nair, 2013); therefore, people aged 60 and above were selected to conduct a more accurate analysis of the research subjects. Finally, the selected studies had no language restrictions, avoiding publication bias as much as possible, all articles were randomized with controlled designs, and the measurement methods of OS indicators were objective, making the evidence obtained more authentic and credible.

Several limitations should be noted when considering the results. First, although the OS indicators in the blood were selected, the analysis of the resulting data was significantly heterogeneous due to different collection and measurement methods. Second, the recruited participants among the included studies were not completely homogeneous (such as healthy people, high blood pressure, obesity, and end-stage renal disease) and were also a potential factor for the difference in results. Third, the duration, frequency, and type of aerobic exercise in the experimental group varied widely, which made it difficult to draw accurate and recommendable conclusions. Fourth, six indicators, including Ox-LDL, 8-isoPGF2, 3-NT, VE, GSH/GSSG, and GSH, were measured in only one study, so the pooled effect could not be analyzed. Finally, there may have been potential reporting bias in this systematic review, because its protocol has not been registered previously. In addition, the sample size of nine included studies was less than 50, which may have caused a type II error (Marchi and Bianchi, 1992); additionally, type I errors should not be ignored due to potential systematic bias. Therefore, the evidence in this review should be interpreted with caution.

## Conclusion

The current systematic review and meta-analysis reveal that regular aerobic exercise may be effective for reducing oxidant marker levels (MDA, LPO, 8-OhdG, and 8-isoPGF2) or increasing antioxidant marker levels (TAC, NO, SOD, VE, and GSH/GSSG). However, considering the significant heterogeneity among studies, these findings should be interpreted with caution.

## Data Availability Statement

The raw data supporting the conclusions of this article will be made available by the authors, without undue reservation.

## Author Contributions

LC, GZ, and JT conceived and designed the study. YY and PQ participated in the literature search. YY and HL contributed to the analyses. YY and MW completed the methodological assessment. YY and GZ analyzed the data and wrote the manuscript. RX and JH were invited to comment and contribute changes. All authors contributed to the article and approved the submitted version.

## Funding

The work was supported by National Natural Science Foundation of China (Grant No. 82074510).

## Conflict of Interest

The authors declare that the research was conducted in the absence of any commercial or financial relationships that could be construed as a potential conflict of interest.

## Publisher’s Note

All claims expressed in this article are solely those of the authors and do not necessarily represent those of their affiliated organizations, or those of the publisher, the editors and the reviewers. Any product that may be evaluated in this article, or claim that may be made by its manufacturer, is not guaranteed or endorsed by the publisher.
