# Supplementary material for: The Effects of Aerobic Exercise on Oxidative Stress in Older Adults: A Systematic Review and Meta-Analysis
Source: Front Physiol. 2021 Oct 5;12:701151. doi: 10.3389/fphys.2021.701151 (PMC8523805; doi:10.3389/fphys.2021.701151)
Supplement: Supplementary file 1 [file Data_Sheet_1.docx]

Supplementary Material

**Search Strategies**

**PubMed**

#1 exercise [ALL]

#2 fast walking [ALL]

#3 tai ji [ALL]

#4 taijiquan [ALL]

#5 tai chi [ALL]

#6 jogging [ALL]

#7 yoga [ALL]

#8 brisk walking [ALL]

#9 qigong [ALL]

#10 physical activity [ALL]

#11 physical endurance [ALL]

#12 treadmill [ALL]

#13 bicycling [ALL]

#14 dance [ALL]

#15 rope skipping [ALL]

#16 physical fitness [ALL]

#17 physical exertion [ALL]

#18 Physical Therapy Modalities [ALL]

#19 endurance activity [ALL]

#20 cardio activity [ALL]

#21 kinesiotherapy [ALL]

#22 swim [ALL]

#23 row [ALL]

#24 skate [ALL]

#25 walk [ALL]

#26 running [ALL]

#27 weight train [ALL]

#28 muscle strength [ALL]

#29 baduanjin [ALL]

#30 ba duan jin [ALL]

#31 sport [ALL]

#32 motion [ALL]

#33 bike [ALL]

#34 cycle [ALL]

#35 #1—#34/OR

#36 older adults[ALL]

#37 elderly [ALL]

#38 older people [ALL]

#39 aging[ALL]

#40 geriatric [ALL]

#41 #36—#40/OR

#42 controlled trial [ALL]

#43 comparison [ALL]

#44 randomized controlled trial [ALL]

#45 #42—#44/OR

#46 oxidative stress [ALL]

#47 oxidation reduction [ALL]

#48 redox* [ALL]

#49 redox stimulus induces [ALL]

#50 redox state [ALL]

#51 reductive stress [ALL]

#52 redox biomarkers [ALL]

#53 redox parameters [ALL]

#54 oxidat* [ALL]

#55 antioxidat* [ALL]

#56 pro-oxidant [ALL]

#57 free radicals [ALL]

#58 active oxygen [ALL]

#59 reactive oxygen species [ALL]

#60 oxygen radicals [ALL]

#61 ROS [ALL]

#62 reactive nitrogen species [ALL]

#63 species reactive nitrogen [ALL]

#64 RNS [ALL]

#65 #46—#64/OR

#66 #35 AND #41 AND #45 AND #65

**Cochrane**

#1 exercise

#2 fast walking

#3 tai ji

#4 taijiquan

#5 tai chi

#6 jogging

#7 yoga

#8 brisk walking

#9 qigong

#10 physical activity

#11 physical endurance

#12 treadmill

#13 bicycling

#14 dance

#15 rope skipping

#16 physical fitness

#17 physical exertion

#18 Physical Therapy Modalities

#19 endurance activity

#20 cardio activity

#21 kinesiotherapy

#22 swim

#23 row

#24 skate

#25 walk

#26 running

#27 weight training

#28 muscle strength

#29 baduanjin

#30 ba duan jin

#31 sport

#32 motion

#33 bike

#34 cycle

#35 #1—#34/OR

#36 older adults

#37 elderly

#38 older people

#39 aging

#40 geriatric

#41 #36—#40/OR

#42 controlled trial

#43 comparison

#44 randomized controlled trial

#45 #42—#44/OR

#46 oxidative stress

#47 oxidation reduction

#48 redox*

#49 redox stimulus induces

#50 redox state

#51 reductive stress

#52 redox biomarkers

#53 redox parameters

#54 oxidat*

#55 antioxidat*

#56 pro-oxidant

#57 free radicals

#58 active oxygen

#59 reactive oxygen species

#60 oxygen radicals

#61 ROS

#62 reactive nitrogen species

#63 species reactive nitrogen

#64 RNS

#65 #46—#64/OR

#66 #35 AND #41 AND #45 AND #65

**Embase**

#1 'exercise'/exp OR exercise

#2 'fast walking' OR (fast AND ('walking'/exp OR walking))

#3 'tai ji'/exp OR 'tai ji' OR (tai AND ji)

#4 jogging

#5 yoga

#6 'brisk walking'

#7 qigong

#8 'physical activity'/exp OR 'physical activity'

#9 'physical endurance'/exp OR 'physical endurance'

#10 treadmill

#11 'bicycling'/exp OR bicycling

#12 'dance'/exp OR dance

#13 'rope skipping' OR (rope AND ('skipping'/exp OR skipping))

#14 'physical fitness'/exp OR 'physical fitness'

#15 'physical exertion'/exp OR 'physical exertion'

#16 'physical therapy modalities'/exp OR 'physical therapy modalities'

#17 'endurance activity'

#18 'cardio activity'

#19 'kinesiotherapy'/exp OR 'kinesiotherapy'

#20 swim

#21 row

#22 skate

#23 walk

#24 running

#25 'weight training'/exp OR 'weight training'

#26 'muscle strength'

#27 baduanjin

#28 'ba duan jin'

#29 'sport'/exp OR sport

#30 'motion'/exp OR motion

#31 bike

#32 cycle

#33 #1—#32/OR 2567837

#34 'older adults'

#35 'elderly'/exp OR elderly

#36 'older people'/exp OR 'older people'

#37 'aging'/exp OR aging

#38 geriatric

#39 #34—#38/OR 3773070

#40 'controlled trial'/exp OR 'controlled trial'

#41 'comparison'/exp OR comparison

#42 'randomized controlled trial'/exp OR 'randomized controlled trial'

#43 #40—#42/OR 9531125

#44 'oxidative stress'/exp OR 'oxidative stress' OR (oxidative AND ('stress'/exp OR stress))

#45 'oxidation reduction'/exp OR 'oxidation reduction' OR (('oxidation'/exp OR oxidation) AND ('reduction'/exp OR reduction))

#46 redox*

#47 'redox stimulus induces'

#48 'redox state'/exp OR 'redox state'

#49 'reductive stress'

#50 oxidat*

#51 antioxidat*

#52 'pro-oxidant'

#53 'free radicals'/exp OR 'free radicals'

#54 'active oxygen'

#55 'reactive oxygen species'

#56 ROS

#57 'oxygen radicals'

#58 'reactive nitrogen species'

#59 RNS

#60 'species reactive nitrogen'

#61 #44—#60/OR 1019860

#62 #33 AND #39 AND #43 AND #61

**Web of Science**

#1 TS=（exercise）

#2 TS=（fast walking）

#3 TS=（tai ji）

#4 TS=（taijiquan）

#5 TS=（tai chi）

#6 TS=（jogging）

#7 TS=（yoga）

#8 TS=（brisk walking）

#9 TS=（qigong）

#10 TS=（physical activity）

#11 TS=（physical endurance）

#12 TS=（treadmill）

#13 TS=（bicycling）

#14 TS=（dance）

#15 TS=（rope skipping）

#16 TS=（physical fitness）

#17 TS=（physical exertion）

#18 TS=（physical therapy modalities）

#19 TS=（endurance activity）

#20 TS=（cardio activity）

#21 TS=（kinesiotherapy）

#22 TS=（swim）

#23 TS=（row）

#24 TS=（skate）

#25 TS=（walk）

#26 TS=（running）

#27 TS=（weight train）

#28 TS=（muscle strength）

#29 TS=（ba duan jin）

#30 TS=（baduanjin）

#31 TS=（sport）

#32 TS=（motion）

#33 TS=（bike）

#34 TS=（cycle）

#35 #1—#34/OR

#36 TS=（older adults）

#37 TS=（elderly）

#38 TS=（older people）

#39 TS=（aging）

#40 TS=（geriatric）

#41 #36—#40/OR

#42 TS=（controlled trial）

#43 TS=（comparison）

#44 TS=（randomized controlled trial）

#45 #42—#44/OR

#46 TS=（oxidative stress）

#47 TS=（oxidation reduction）

#48 TS=（redox*）

#49 TS=（redox stimulus induces）

#50 TS=（redox state）

#51 TS=（reductive stress）

#52 TS=（redox biomarkers）

#53 TS=（redox parameters）

#54 TS=（oxidat* ）

#55 TS=（antioxidat* ）

#56 TS=（prooxidant）

#57 TS=（free radicals）

#58 TS=（active oxygen）

#59 TS=（reactive oxygen species）

#60 TS=（oxygen radicals）

#61 TS=（ROS）

#62 TS=（reactive nitrogen species）

#63 TS=（species reactive nitrogen）

#64 TS=（RNS）

#65 #46—#64/OR

#66 #35 AND #41 AND #45 AND #65

**SinoMed**

#1 运动[全部字段]

#2 体育锻炼[全部字段]

#3 体育活动[全部字段]

#4 耐力运动[全部字段]

#5 耐力活动[全部字段]

#6 耐力训练[全部字段]

#7 运动疗法[全部字段]

#8 物理治疗[全部字段]

#9 心肺训练[全部字段]

#10 重力训练[全部字段]

#11 肌肉力量[全部字段]

#12 步行[全部字段]

#13 快步走[全部字段]

#14 健身走[全部字段]

#15 慢跑[全部字段]

#16 跑步[全部字段]

#17 跑步机[全部字段]

#18 骑车[全部字段]

#19 自行车[全部字段]

#20 骑行[全部字段]

#21 跳绳[全部字段]

#22 跳舞[全部字段]

#23 游泳[全部字段]

#24 滑冰[全部字段]

#25 瑜伽[全部字段]

#26 气功[全部字段]

#27 太极[全部字段]

#28 八段锦[全部字段]

#29 ＃1-＃28 / OR

#30 老年[全部字段]

#31 老年人[全部字段]

#32 老人[全部字段]

#33 衰老[全部字段]

#34 老龄化[全部字段]

#35 ＃30-＃34 / OR

#36 对照[全部字段]

#37 对照试验[全部字段]

#38 随机对照试验[全部字段]

#39 ＃36-＃38 / OR

#40 氧化[全部字段]

#41 氧化剂[全部字段]

#42 抗氧化酶[全部字段]

#43 抗氧化剂[全部字段]

#44 还原酶[全部字段]

#45 还原剂[全部字段]

#46 自由基[全部字段]

#47 活性氧[全部字段]

#48 活性氮[全部字段]

#49 ROS[全部字段]

#50 RNS[全部字段]

#51 ＃40-＃50 / OR

#52 #29 AND #35 AND #39 AND #51

**China Science and Technology Journal Database (VIP)**

#1 U=运动

#2 U=体育锻炼

#3 U=体育活动

#4 U=耐力运动

#5 U=耐力活动

#6 U=耐力训练

#7 U=运动疗法

#8 U=物理治疗

#9 U=心肺训练

#10 U=重力训练

#11 U=肌肉力量

#12 U=步行

#13 U=快步走

#14 U=健身走

#15 U=慢跑

#16 U=跑步

#17 U=跑步机

#18 U=骑车

#19 U=自行车

#20 U=骑行

#21 U=跳绳

#22 U=跳舞

#23 U=游泳

#24 U=滑冰

#25 U=瑜伽

#26 U=气功

#27 U=太极

#28 U=八段锦

#29 U=＃1-＃28 / OR

#30 U=老年

#31 U=老年人

#32 U=老人

#33 U=衰老

#34 U=老龄化

#35 U=＃30-＃34 / OR

#36 U=氧化

#37 U=氧化剂

#38 U=抗氧化酶

#39 U=抗氧化剂

#40 U=还原酶

#41 U=还原剂

#42 U=自由基

#43 U=活性氧

#44 U=活性氮

#45 U=ROS

#46 U=RNS

#47 U=＃36-＃46 / OR

#48 #29 AND #35 AND #47

**Wanfang Date**

#1 运动

#2 体育锻炼

#3 体育活动

#4 耐力运动

#5 耐力活动

#6 耐力训练

#7 运动疗法

#8 物理治疗

#9 心肺训练

#10 重力训练

#11 肌肉力量

#12 步行

#13 快步走

#14 健身走

#15 慢跑

#16 跑步

#17 跑步机

#18 骑车

#19 自行车

#20 骑行

#21 跳绳

#22 跳舞

#23 游泳

#24 滑冰

#25 瑜伽

#26 气功

#27 太极

#28 八段锦

#29 ＃1-＃28 / OR

#30 老年

#31 老年人

#32 老人

#33 衰老

#34 老龄化

#35 ＃30-＃34 / OR

#36 对照

#37 对照试验

#38 随机对照试验

#39 ＃36-＃38 / OR

#40 氧化

#41 氧化剂

#42 抗氧化酶

#43 抗氧化剂

#44 还原酶

#45 还原剂

#46 自由基

#47 活性氧

#48 活性氮

#49 ROS

#50 RNS

#51 ＃40-＃50 / OR

#52 #29 AND #35 AND #39 AND #51

**China National Knowledge Infrastructure (CNKI)**

#1 AB=运动

#2 AB=体育锻炼

#3 AB=体育活动

#4 AB=耐力运动

#5 AB=耐力活动

#6 AB=耐力训练

#7 AB=运动疗法

#8 AB=物理治疗

#9 AB=心肺训练

#10 AB=重力训练

#11 AB=肌肉力量

#12 AB=步行

#13 AB=快步走

#14 AB=健身走

#15 AB=慢跑

#16 AB=跑步

#17 AB=跑步机

#18 AB=骑车

#19 AB=自行车

#20 AB=骑行

#21 AB=跳绳

#22 AB=跳舞

#23 AB=游泳

#24 AB=滑冰

#25 AB=瑜伽

#26 AB=气功

#27 AB=太极

#28 AB=八段锦

#29 ＃1-＃28 / OR

#30 AB=老年

#31 AB=老年人

#32 AB=老人

#33 AB=衰老

#34 AB=老龄化

#35 ＃30-＃34 / OR

#36 AB=氧化

#37 AB=氧化剂

#38 AB=抗氧化酶

#39 AB=抗氧化剂

#40 AB=还原酶

#41 AB=还原剂

#42 AB=自由基

#43 AB=活性氧

#44 AB=活性氮

#45 AB=ROS

#46 AB=RNS

#47 ＃36-＃46 / OR

#52 #29 AND #35 AND #47
