# Supplementary material for: The Effects of Aerobic Exercise on Oxidative Stress in Older Adults: A Systematic Review and Meta-Analysis
Source: Front Physiol. 2021 Oct 5;12:701151. doi: 10.3389/fphys.2021.701151 (PMC8523805; doi:10.3389/fphys.2021.701151)

Supplementary Material

**Evaluation of oxidative stress markers effects shown by forest plots**

1. **Pro-oxidant Markers**

1.1 MDA


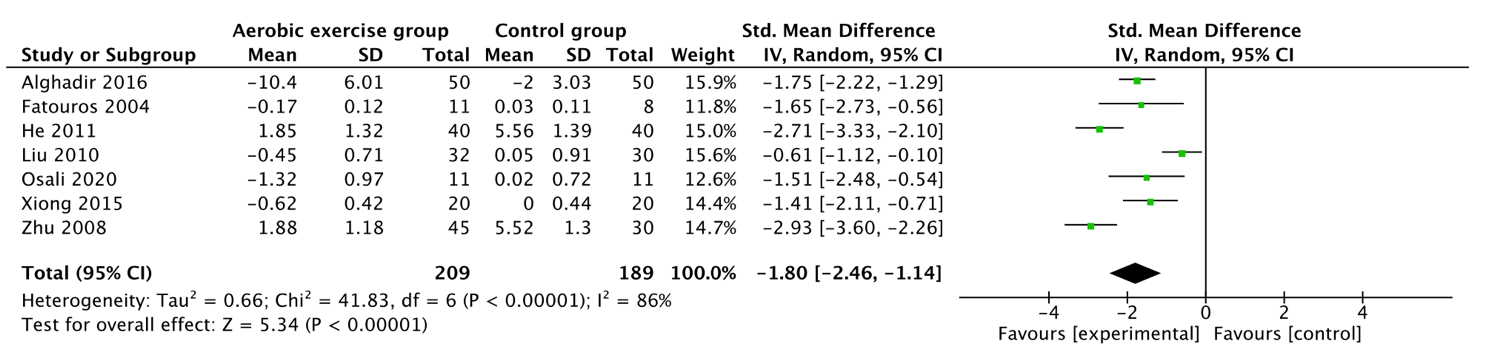


- 1. 8-OHdG

**
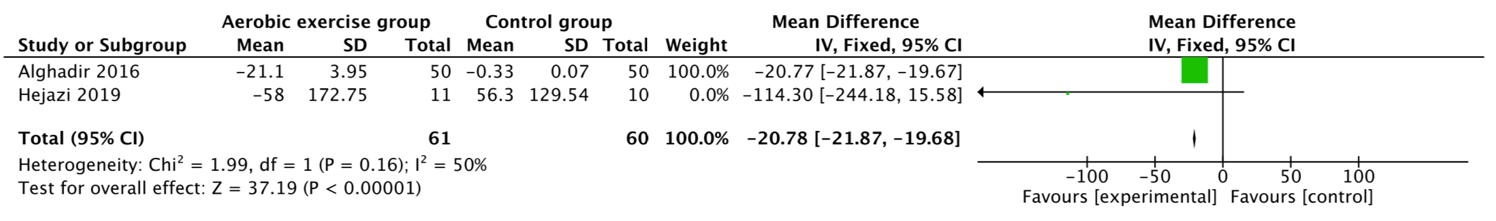
**

- 1. LPO


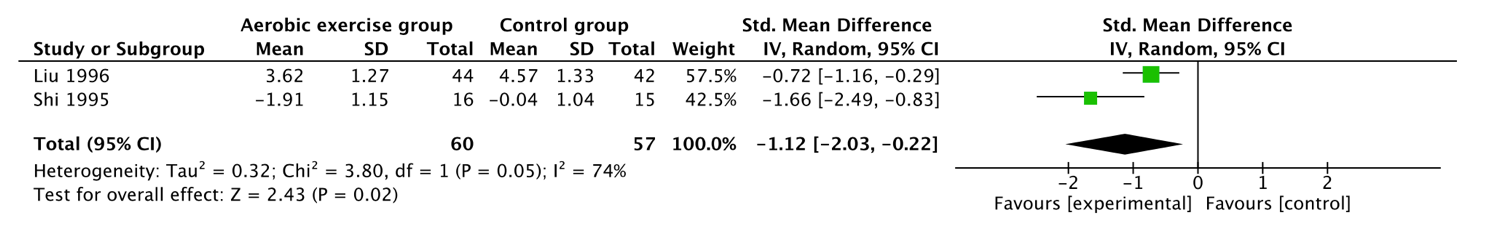


- 1. 3-NT


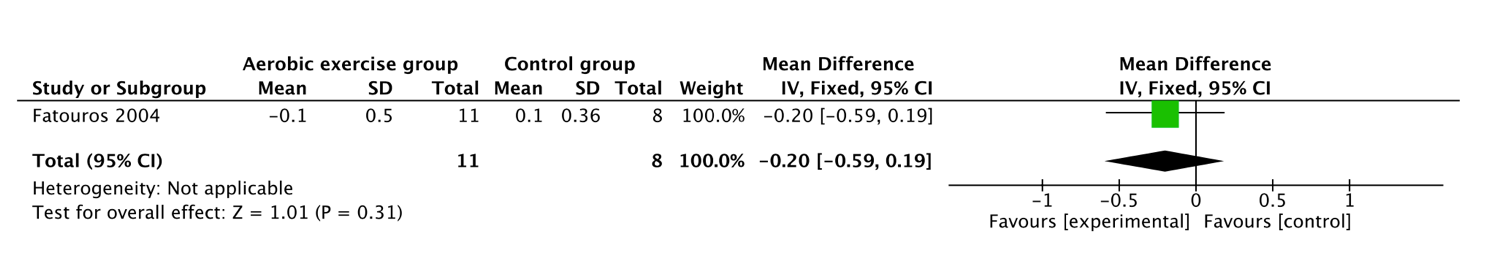


- 1. Ox-LDL


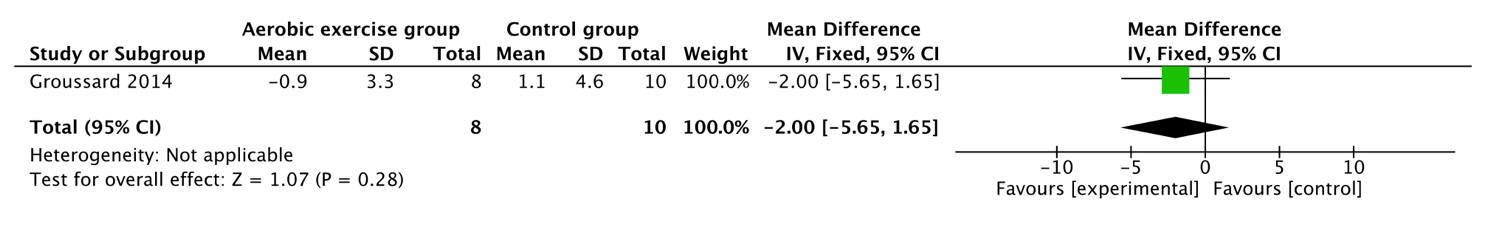


- 1. 8-isoPGF2


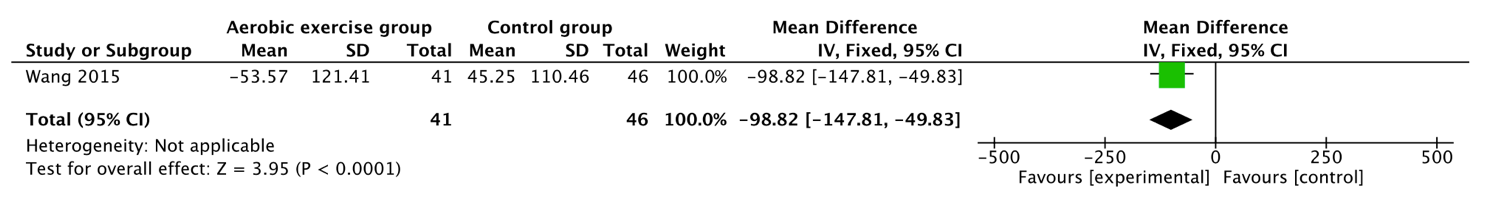


1. **Antioxidant Markers**

2.1 NO


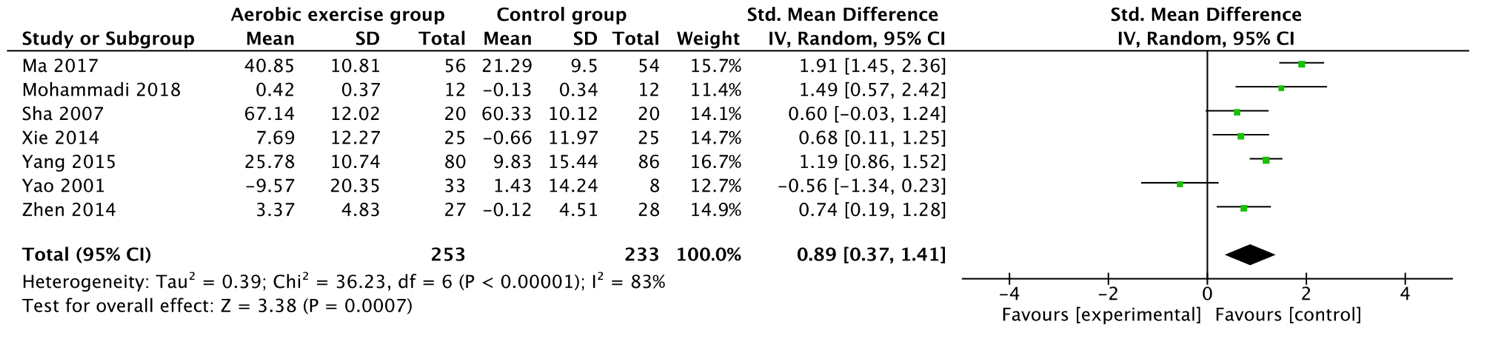


- 1. SOD


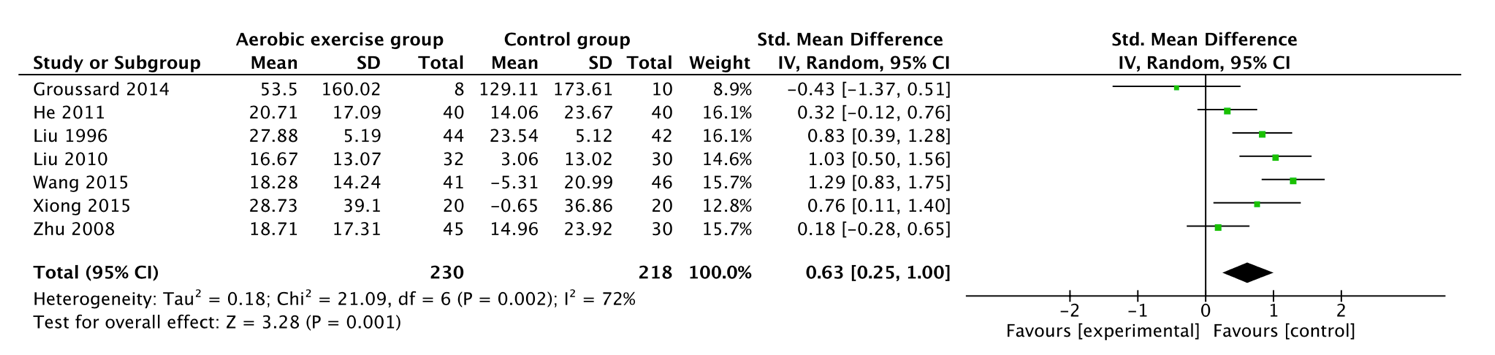


- 1. TAC


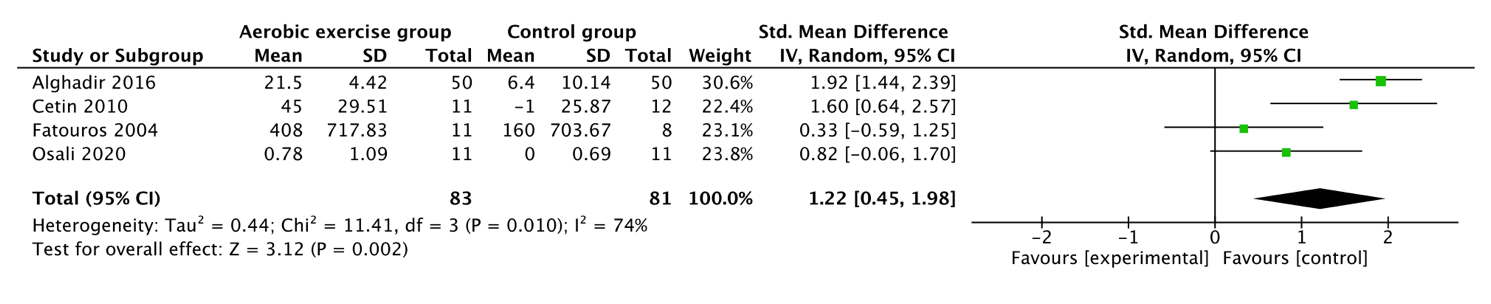


- 1. GPX


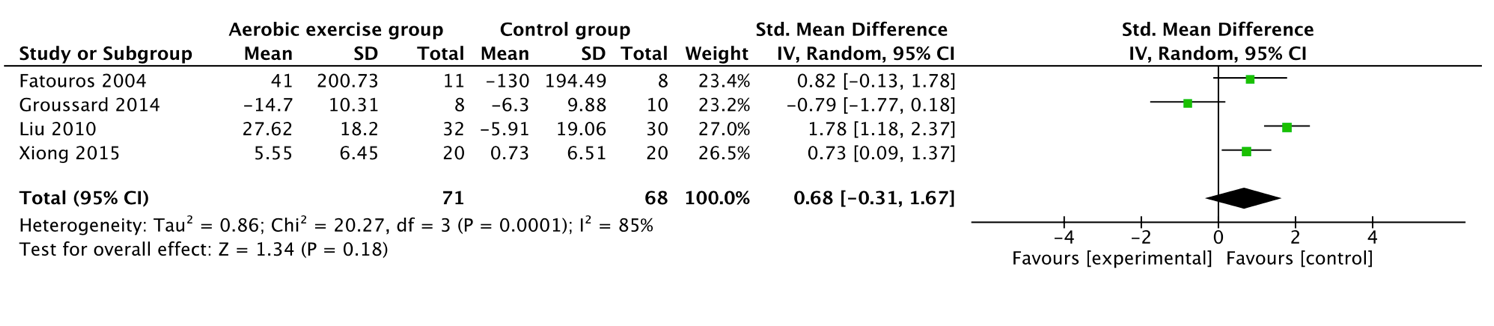


- 1. GSH


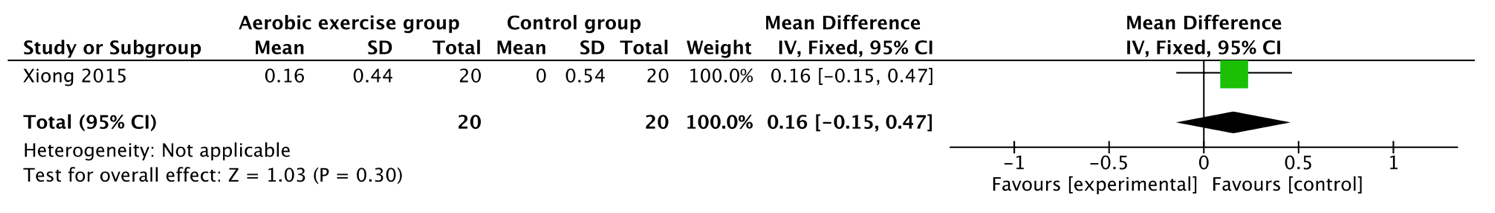


- 1. Vitamin E

**
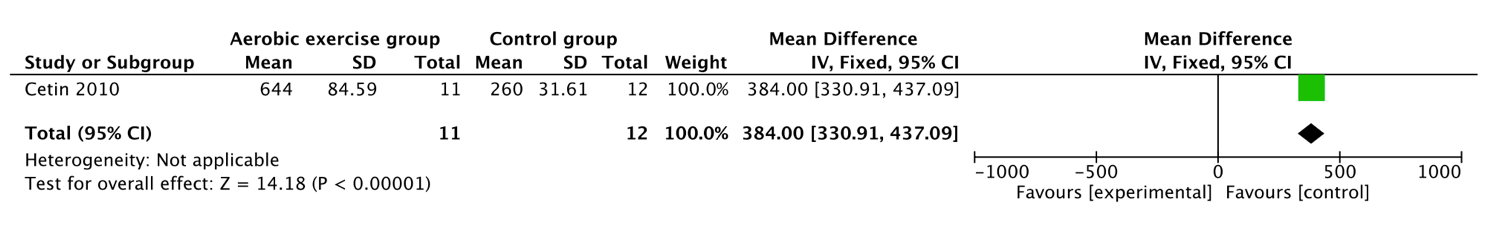
**

2.7 GSH/GSSG

**
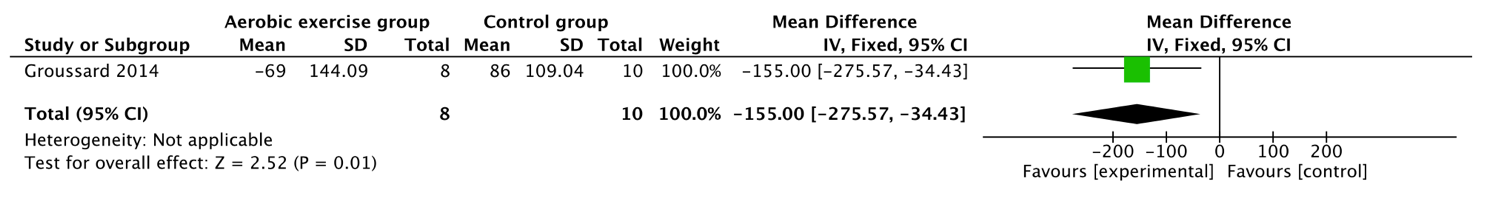
**

1. **Subgroup analysis**

3.1 MDA

**
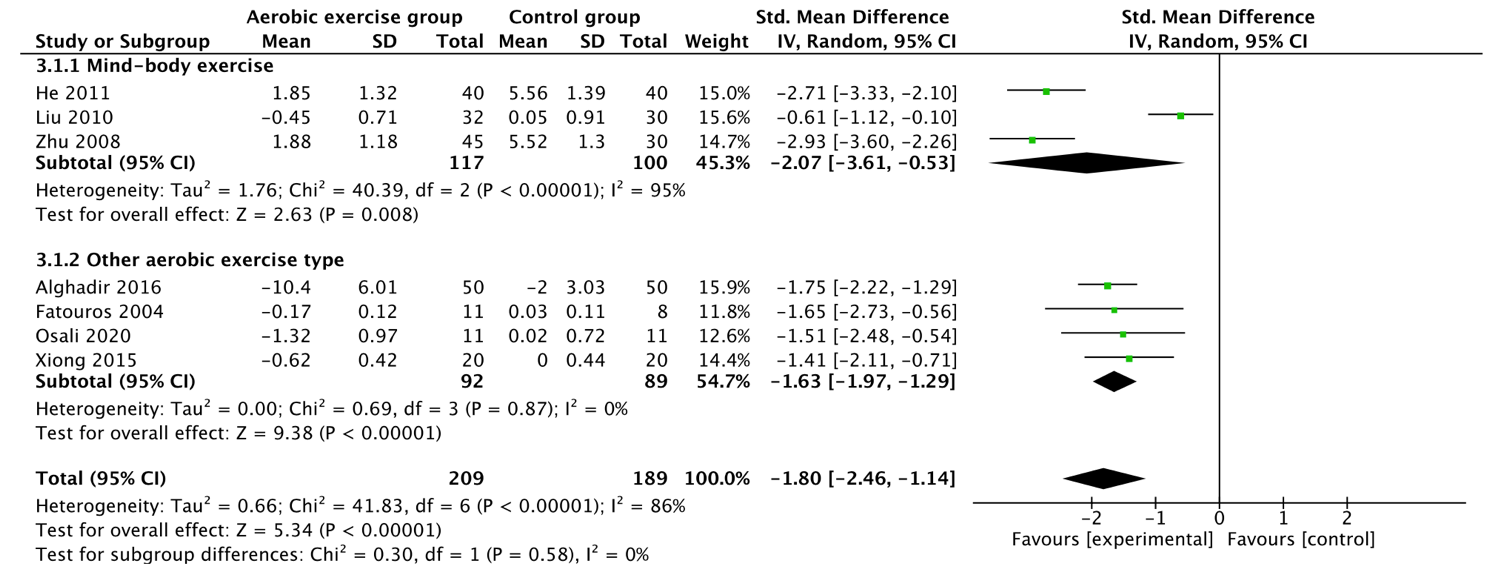
**

- 1. NO

**
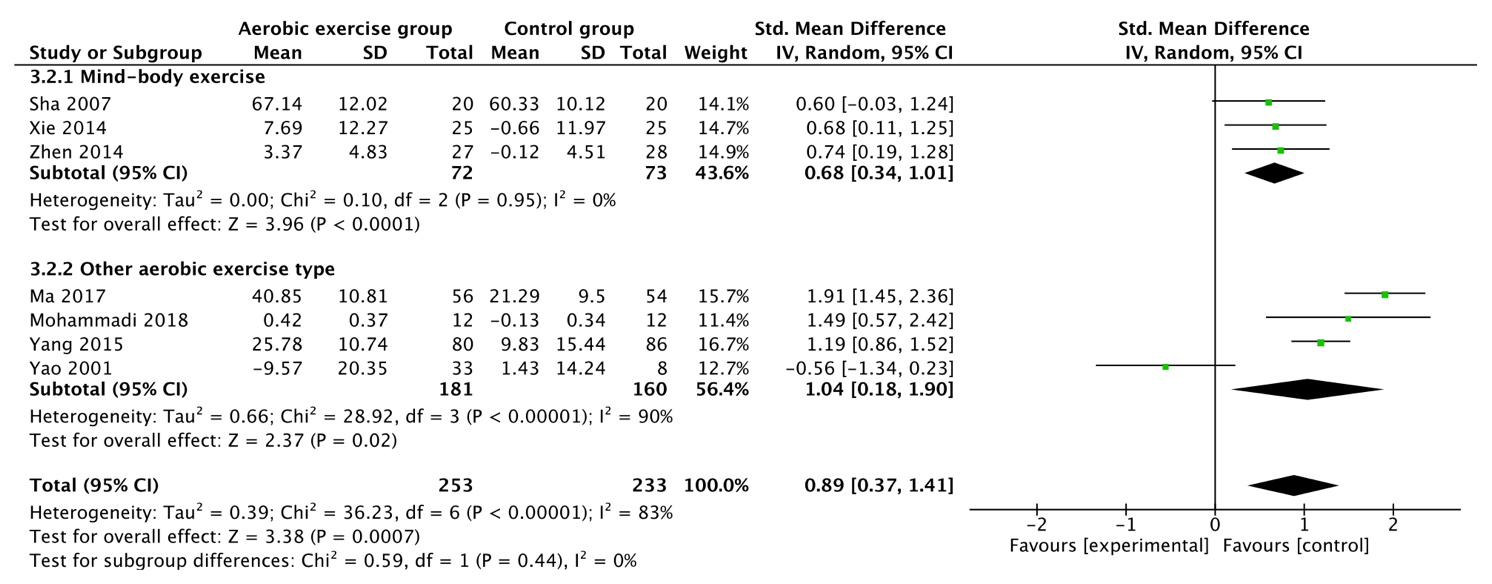
**

**
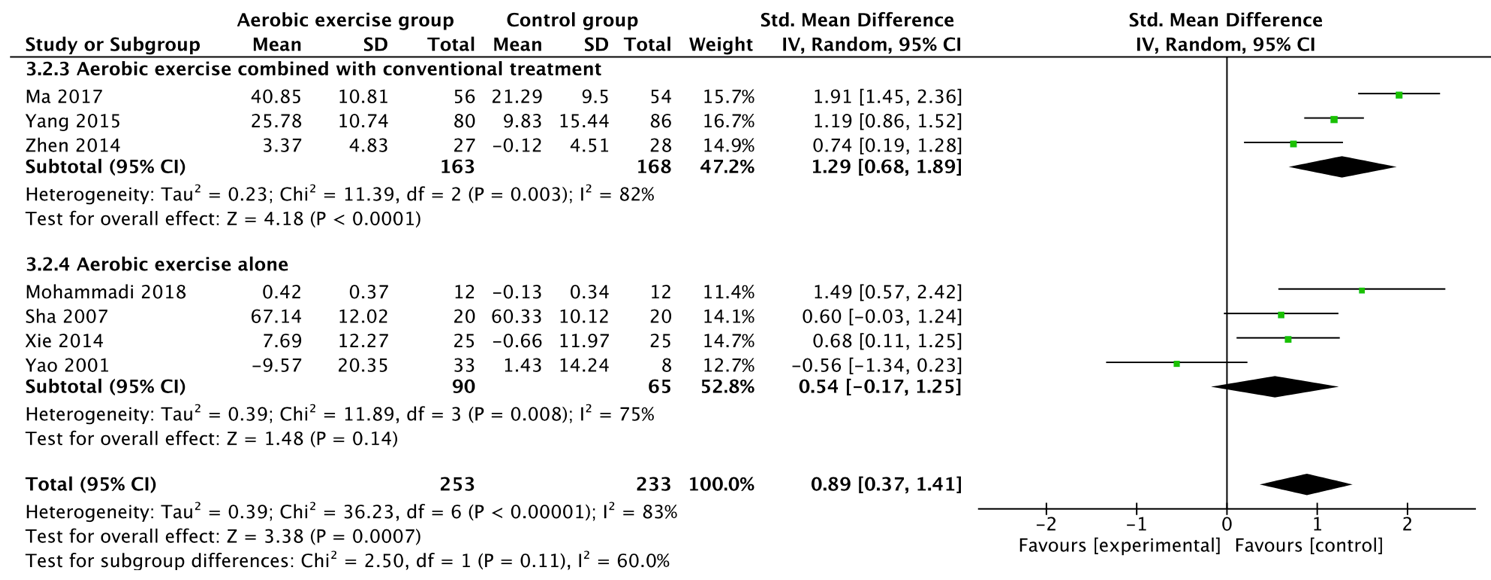
**

- 1. SOD

**
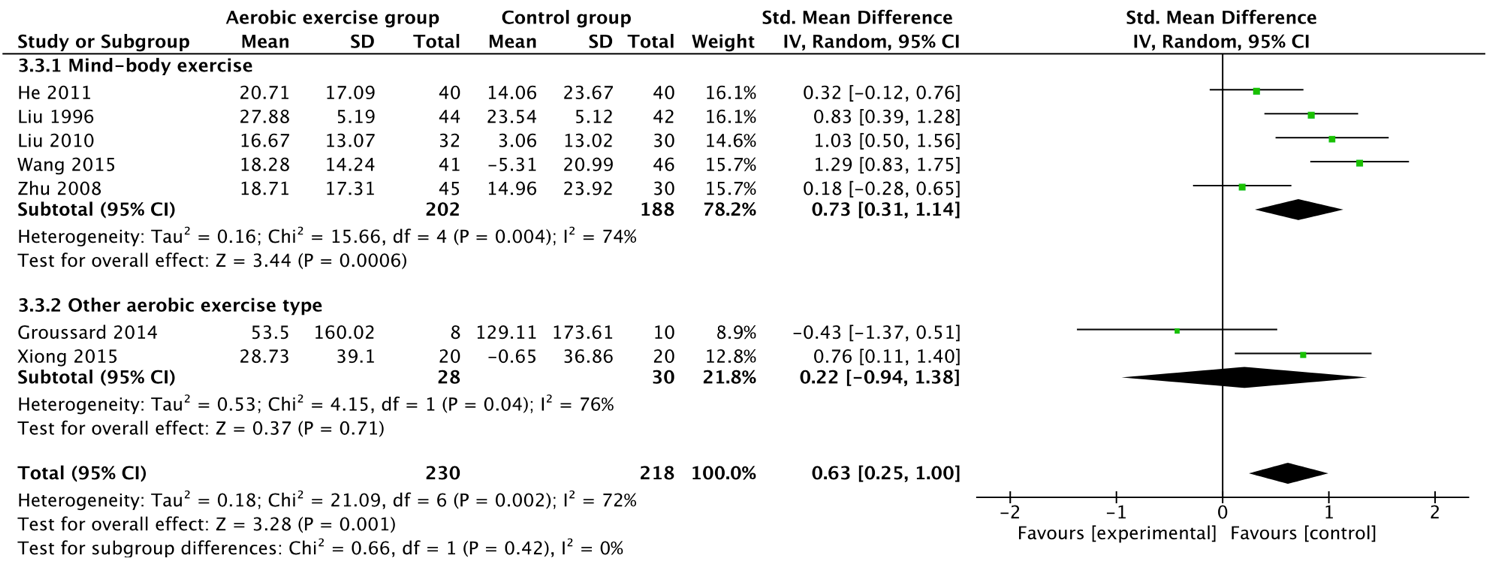
**

1. **Sensitivity analysis for the subgroup**

4.1 NO


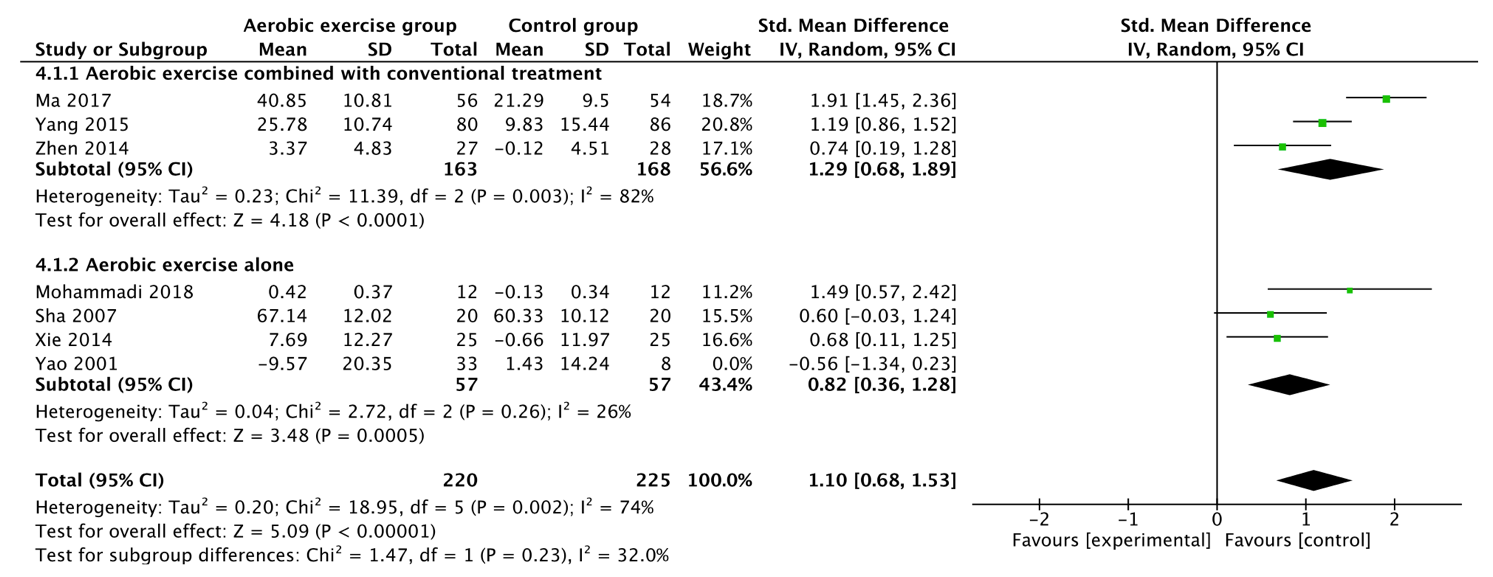

Supplement: Supplementary file 2 [file Data_Sheet_2.docx]
